# QTL mapping of adult plant and seedling resistance to leaf rust (*Puccinia triticina* Eriks.) in a multiparent advanced generation intercross (MAGIC) wheat population

**DOI:** 10.1007/s00122-020-03657-2

**Published:** 2020-08-19

**Authors:** Sandra Rollar, Albrecht Serfling, Manuel Geyer, Lorenz Hartl, Volker Mohler, Frank Ordon

**Affiliations:** 1 Institute for Resistance Research and Stress Tolerance, Julius Kuehn-Institute, Erwin Baur‑Straße 27, 06484 Quedlinburg, Germany; 2 Bavarian State Research Center for Agriculture, Institute for Crop Science and Plant Breeding, Am Gereuth 8, Freising, Germany

## Abstract

**Key message:**

The Bavarian MAGIC Wheat population, comprising 394 F6:8 recombinant inbred lines was phenotyped for Puccinia triticina resistance in multi-years’ field trials at three locations and in a controlled environment seedling test. Simple intervall mapping revealed 19 QTL, corresponding to 11 distinct chromosomal regions.

**Abstract:**

The biotrophic rust fungus *Puccinia triticina* is one of the most important wheat pathogens with the potential to cause yield losses up to 70%. Growing resistant cultivars is the most cost-effective and environmentally friendly way to encounter this problem. The emergence of leaf rust races being virulent against common resistance genes increases the demand for wheat varieties with novel resistances. In the past decade, the use of complex experimental populations, like multiparent advanced generation intercross (MAGIC) populations, has risen and offers great advantages for mapping resistances. The genetic diversity of multiple parents, which has been recombined over several generations, leads to a broad phenotypic diversity, suitable for high-resolution mapping of quantitative traits. In this study, interval mapping was performed to map quantitative trait loci (QTL) for leaf rust resistance in the Bavarian MAGIC Wheat population, comprising 394 F_6:8_ recombinant inbred lines (RILs). Phenotypic evaluation of the RILs for adult plant resistance was carried out in field trials at three locations and two years, as well as in a controlled-environment seedling inoculation test. In total, interval mapping revealed 19 QTL, which corresponded to 11 distinct chromosomal regions controlling leaf rust resistance. Six of these regions may represent putative new QTL. Due to the elite parental material, RILs identified to be resistant to leaf rust can be easily introduced in breeding programs.

**Electronic supplementary material:**

The online version of this article (10.1007/s00122-020-03657-2) contains supplementary material, which is available to authorized users.

## Introduction

With approximately 219 million hectares worldwide and 30% of global major cereal crop production in 2017, wheat (*Triticum* spp.) belongs to the most important crops for human nutrition (Braun et al. 2010; FAO 2019). Leaf rust, caused by the obligate biotrophic fungus *Puccinia triticina* Eriks., is nowadays the most destructive and prevalent rust pathogen in wheat (Kolmer 2005). Due to its adaptation to a wide range of different environments, leaf rust occurs in many wheat-producing areas of the temperate zone, causing yield losses up to 70% (Aktar-Uz-Zaman et al. 2017; Herrera-Foessel et al. 2006; Marasas et al. 2004). Although the application of fungicides helps to avoid yield losses, the deployment of resistant cultivars is the most effective, economic, and environmentally friendly approach to manage this disease. For wheat leaf rust, both qualitative and quantitative resistances are known. Seedling/qualitative resistance is monogenically inherited and only effective against a subset of races. Thus, it mainly follows the gene-for-gene concept, in which resistance depends on a specific genetic interaction between host-resistance genes and avirulence genes of the pathogen (Flor 1956, 1971). These major genes confer vertical resistance and tend to be expressed from seedling to adult plant stages. Genotypes carrying such resistances show a hypersensitive response or programmed cell death (Bolton et al. 2008). In contrast, quantitative resistance is based on minor genes encoding various resistance responses, which are not restricted to specific pathogen races. Quantitative resistances are effective at later growth stages and are therefore referred to as field resistance or adult plant resistance (APR, Krattinger and Keller 2016). To date, more than 80 resistance genes to leaf rust (*Lr* genes) have been identified in bread wheat, durum wheat, and diploid wheat species (Gill et al. 2019). While most of them show race-specific resistance at the seedling stage, genes like *Lr12*, *Lr13*, *Lr22a/b*, *Lr34*, *Lr35*, *Lr37*, *Lr46*, *Lr67*, *Lr68*, and *Lr77* confer resistance at the adult plant stage (Dakouri et al. 2013; McIntosh et al. 2013, 2017).

The identification of such resistance genes as well as of quantitative trait loci (QTL) has been mainly based on biparental crosses (Huang et al. 2012). The weakness of such populations is the narrow genetic variation and the fact that genetic recombination is limited, which leads to a lower map resolution (Bandillo et al. 2013). Nowadays, high-throughput marker systems are available and genetic marker information is no longer limiting (Bayer et al. 2017; Chen et al. 2014; Cui et al. 2017; He et al. 2014; Mammadov et al. 2012), but the genetic variation present in respective populations (Asimit and Zeggini 2010; Gibson 2012). Thus, complex experimental populations such as nested association mapping (NAM, Yu et al. 2008) and multiparent advanced generation intercross (MAGIC) populations have been developed to detect QTL with a better reliability (Cavanagh et al. 2008). First multiparental intermated populations were exploited in mice (Churchill et al. 2004) and *Drosophila melanogaster* (King et al. 2012). In plants, MAGIC populations were first developed and described in studies regarding *Arabidopsis thaliana* (Cavanagh et al. 2008; Kover et al. 2009). These experimental designs involved multiple intercrosses of inbred founders for several generations to combine the genetic variation of all parental lines in the resulting progeny (Huang et al. 2012). MAGIC populations have been widely used to conduct QTL mapping in several crop species, such as rice (Bandillo et al. 2013), maize (Dell’Acqua et al. 2015), tomato (Pascual et al. 2015), faba bean (Sallam and Martsch 2015), sorghum (Ongom and Ejeta 2018), barley (Sannemann et al. 2015), and wheat (Gardner et al. 2016; Huang et al. 2012; Mackay et al. 2014; Milner et al. 2016; Sannemann et al. 2018). There are two clear advantages of using multiparental populations. First, based on the choice of founders, more traits of interest from each founder can be analyzed. Second, due to the higher genetic variability and recombination rate, QTL detection can be performed with increased precision and resolution (Bandillo et al. 2013; Cavanagh et al. 2008).

The Bavarian MAGIC Wheat population (BMWpop) is one of only two German MAGIC wheat populations, which are mainly based on adapted German elite cultivars. It captures 71.7% of the allelic diversity available in the German wheat breeding gene pool (Stadlmeier et al. 2018). These populations provide the potential to carry out genetic studies of important economical traits, such as plant height and resistance to powdery mildew (Sannemann et al. 2018; Stadlmeier et al. 2018). In addition, Stadlmeier et al. (2019) detected six, seven and nine QTL for resistance to important fungal pathogens, i.e., *Blumeria graminis*, *Zymoseptoria tritici*, and *Pyrenophora tritici-repentis*, respectively. The objectives of the current study were to (1) phenotype the BMW population for quantitative and qualitative leaf rust resistance in multi-environment field trials and an extensive seedling test and (2) genetically map QTL in order to develop closely linked molecular markers suitable for marker-assisted selection (MAS).

## Material and methods

### Plant material

The study is based on the multiparental BMW population comprising elite wheat cultivars (Stadlmeier et al. 2018). It consists of 394 diverse F_6:8_ recombinant inbred lines (RILs), which were derived from a simplified eight founder MAGIC mating design with additional eight-way intercrosses. The founders ‘Event’, ‘BAYB4535′, ‘Potenzial’, ‘Bussard’, ‘Firl3565’, ‘Format’, ‘Julius’ and ‘Ambition’ originated from German and Danish wheat breeders and were selected on the criteria of (1) variation for agronomic, quality and disease resistance traits, (2) originating from different breeding programs, and (3) being important cultivars in the respective baking quality group. More detailed information about the development and the genetics of the BMW population is provided by Stadlmeier et al. (2018).

### Phenotypic assessment of leaf rust resistance in field

Five field trials were performed, each using a randomized incomplete block design with two replications at three locations in Germany: Quedlinburg (QLB, 51° 46′ 21.45″ N 11° 8′ 34.8″ E) in Saxony-Anhalt, Soellingen (SOE, 52° 5′ 45.506″ N 10° 55′ 41.711″ E) and Lenglern (LEN, 51° 35′ 47.53″ N 9° 51′ 39.118″ E) in Lower Saxony. The 394 RILs, the eight founders, and the susceptible standard ‘Schamane’ were evaluated in double rows under natural disease epidemics in SOE (2017 and 2018) and LEN (2018). In QLB entries were sown 2016/2017 and 2017/2018 in double rows of 1 m length with 30 plants per row and spacing of 0.2 m between rows. Additional infection stripes of susceptible varieties were arranged in regular intervals of every third plot. Growth regulator Medax® Top (BASF Agricultural Solutions, Germany, 1 L ha^−1^) was applied twice (BBCH31, BBCH37) to reduce plant height and lodging. No selective fungicides were used. To ensure uniform infestation, the infection stripes were artificially inoculated at the beginning of flowering using the highly virulent *Puccinia triticina* isolate 77WxR (Tab. S1). For this, a spore suspension of 10 mg uredospores in 100 ml Isopar M (ExxonMobil Chemical Company, USA) was applied in a total amount of 10 ml suspension per m^2^, using a hand-held spinning disc sprayer (Bromyard, U.K.). Phenotyping of the trials was carried out by scoring the average percentage of infected leaf area of the second and third youngest leaves in the two rows at two (SOE17, SOE18, LEN18), three (QLB18), and four (QLB17) subsequent dates according to Moll et al. (2010), starting at the time of clearly visible disease symptoms on the infection stripe or the susceptible standard, respectively. A time period of 1 to 2 weeks was chosen between the scorings.

### Phenotypic assessment of leaf rust resistance in seedlings

All RILs, the parental lines, and the susceptible standard ‘Borenos’ were evaluated for resistance at seedling stage in a detached leaf assay (Douchkov et al. 2012). Seedlings were grown in 77-cell trays with mixed potting soil (Gebr. Patzer GmbH Co KG, Sinntal, Germany) using a randomized complete block design with five replications. Water agar (7 g L^−1^) containing 45 mg L^−1^ benzimidazole (Sigma-Aldrich Chemie GmbH, Taufkirchen, Germany), used to delay senescence of leaf segments, was dispensed in 4 × 10 mL aliquots into nonsterile four-well polystyrene plates (8 × 12 x 1 cm, Greiner Bio-One GmbH, Frickenhausen, Germany). Ten days after sowing, when the second leaf was developed, 2.5-cm sections were cut from the middle of the primary leaves and placed into the plates, keeping the randomization. White polytetrafluoroethylene frames (eMachineShop, Mahwah, USA) were used to fix the leaves. Inoculation was performed by an infection tower with three seconds swirling duration and three minutes of settling time (Melching 1967). Due to space restrictions, plates were divided into two infection groups per replication. Each group was inoculated with leaf rust isolate 77WxR using a mixture of 30 mg uredospores and white clay (1:1 w/w, VWR International GmbH, Darmstadt, Germany) after application of a 0.01% Tween 20 (Sigma-Aldrich) solution to support adhesion. For 24 h, the plates were covered by wet cotton paper to support spore germination in the dark and at high humidity. Inoculated leaf segments were subsequently incubated in greenhouse at night/day temperatures of 16 °C/18 °C with additional lighting (16 h/8 h day/night) for ten days. Quantitative scoring was conducted using a high-throughput phenotyping platform (Douchkov et al. 2012). Digital images with a resolution of 20 Megapixel and four wavelengths between 315 and 750 nm (UV, blue, green, and red) were taken automatically from every plate. Subsequently, the leaf area was calculated and compared to the area of uredospore pustules for analyzing the percentage of infected leaf area (Pi) using the software HawkSpex® (Fraunhofer IFF, Magdeburg, Germany). Additionally, all entries were visually evaluated for infection type (IT) using a 0−4 scale (McIntosh et al. 1995). To generate metric data, original IT data were converted to a 0 – 10 linear disease scale, modified according to Zhang et al. (2014) as follows: 0, 0;, − 1,1, + 1, − 2, 2, 2 + , − 3, 3, + 3 were coded as 0, 0.5, 1, 2, 3, 4, 5, 6, 7, 8 and 9, respectively. IT − 4 and 4 were coded as 10 and in case of special annotation code “C” for chlorosis, 0.5 was added to the linear scale.

### Data analysis

The multiple scorings of the percentage of infected leaf area in field trials were taken to calculate the area under the disease progress curve (AUDPC) and the average ordinate (AO, Moll et al. 1996) for each RIL using the following equations:

$$AUDPC = \mathop \sum \limits_{i = 1}^{{N_{i - 1} }} \frac{{(y_{i} + y_{i + 1} )}}{2}*\left( {t_{i + 1} - t_{i} } \right)\quad {\text{and}}\quad AO = \frac{{{\text{AUDPC}}}}{{\text{T}}}$$

where *y*_*i*_ is the disease level at the ith observation, *t*_*i*_ is the time at the ith observation, *N* is the total number of observations and *T* is the total observation time from the first to the last scoring date in days. Out of percentage of infected leaf area, AUDPC, and AO, only AO values were used for subsequent statistical analysis. Different year–location combinations of all trials were referred to as “environment”.

Analyses of all phenotypic data were carried out using *proc mixed* of the software package SAS 9.4 (SAS Institute Inc., NY, USA). In order to apply a mixed linear model, a log_10_ data transformation of the AO, IT, and Pi values was performed. The factors genotype, environment, and the genotype × environment interaction of field data were set as fix effects, while the design effects of replication and block were set as random. To obtain variance components for calculation of the broad sense heritability, all model parameters were set as random. Heritability was estimated on a progeny mean basis according to Hallauer et al. (2010).

For analyzing IT and Pi scores from seedling test the model:

$$y_{ijk} = \mu + g_{i} + r_{j} + l_{k} \left( {r_{j} } \right) + e_{ijk}$$

was used, where *y*_*ijk*_ is the trait observation, *µ* is the overall mean, *g*_*i*_ is the fixed effect of the genotype, *r*_*j*_ is the fixed effect of the replication, *l*_*k*_ is the random effect of the infection group nested in the replication and *e*_*ijk*_ is the random residual error. Variance components were obtained by fitting the genotype as random to calculate the repeatability as the ratio of the genotypic variance and the sum of the genotypic and the residual error variance divided by the number of replications. For each trait, least-square means (lsmeans) were calculated and used for subsequent QTL analysis.

### QTL mapping

The BMW population and the parental lines were genotyped using the 15 K + 5 K Infinium® iSelect® array containing 17,267 single nucleotide polymorphism (SNP) markers (TraitGenetics, Germany). The preparation of genotypic data and the construction of the linkage map used for QTL mapping were described in detail by Stadlmeier et al. (2018). QTL mapping was performed using the R (× 32 3.2.5) package mpMap V2.0.2 (Huang and George 2011; R Core Team 2017). To conduct simple interval mapping (SIM), founder probabilities were calculated using the function ‘mpprob’. These give information about the probability of each locus that the observed genotype was inherited from one of the eight founders and are based on multipoint haplotype probabilities (Broman et al. 2003). To determine the parental origin of an allele, the threshold was set to 0.7. For SIM, a genome-wide significant threshold of α < 0.05 was calculated for each trait. The thresholds were obtained from permutation of phenotypic data with 1000 simulation runs (Churchill and Doerge 1994). QTL detection was performed using the function ‘mpIM’, implemented in the mpMap package (Huang and George 2011). Phenotypic variance explained by individual QTL and additive QTL effects were estimated separately using the categorical allele information of the founders. QTL support intervals were determined using the function ‘supportinterval’ of the mpMap package. A QTL support interval was defined as the map interval surrounding a QTL peak at a − log_10_(*p*) drop of one unit (Huang and George 2011).

In order to compare QTL identified in the present study with previously described QTL, overlapping QTL based on the support interval was merged together. Databases of the Triticeae Toolbox (https://triticeaetoolbox.org/wheat/genotyping/marker_selection.php), GrainGenes (https://wheat.pw.usda.gov/GG3/), as well as CerealsDB (https://www.cerealsdb.uk.net/cerealgenomics/CerealsDB/axiom_download.php) were used to obtain marker information. Physical positions were received by nucleotide BLAST (BLAST-n) of the marker sequences against the reference sequence RefSeq v1.0 using the database of 10 + Genome Project (https://webblast.ipk-gatersleben.de/wheat_ten_genomes/). BLAST hits were considered significant if the percent identity was greater than 95%, and only the best hit was taken if multiple BLAST hits were detected (Gao et al. 2016). The start and end positions of peak marker sequences preceded by the chromosome name were taken to the URGI database to obtain functional gene annotations available from IWGSC (https://wheat-urgi.versailles.inra.fr/Seq-Repository/Annotations). Sequences of the closest related species, *Triticum urartu* (A-genome donor) and *Aegilops tauschii* (D-genome donor), were considered for the detection of orthologous genes.

## Results

### Phenotypic assessment

**Fig. 1:**
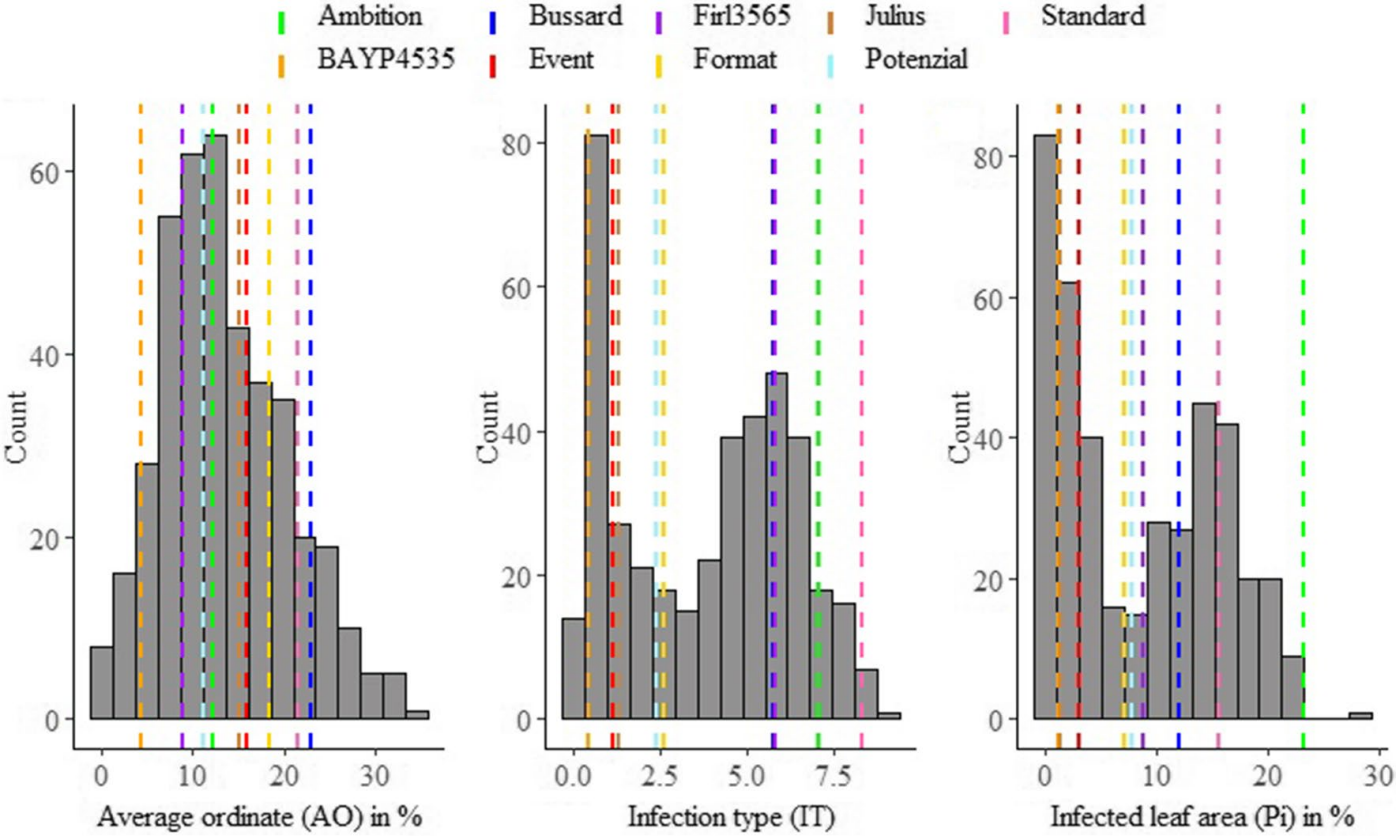
Averaged phenotypic distribution of resistance to *Puccinia triticina* for field trials (A) and seedling test (B, C). Performance of each parental line is shown as vertical dashed line

Leaf rust severity of field trials clearly varied between years and location, displaying in QLB 2017, SOE 2018, and LEN 2018 the lowest infestations of leaf rust (Fig. S1). Pearson correlation coefficient between the different environments ranged from 0.26 to 0.74 (*P* < 0.001). Nevertheless, after mixed model adjustment, a broad sense heritability (*h*^2^) of 0.83 was estimated (Table 1). The mean phenotypic distribution of AOs was slightly right-skewed and indicated a broad variability within the population (Fig. 1a), ranging between 0.2 and 34.8% (mean 13.5%) leaf area diseased. However, single maximal AO scores up to 63.8% were observed within the population (Table 1). The average performance of parental lines was evenly distributed, resulting in a nonsignificant difference (*p* < 0.05) from the progeny mean. Founders ‘BAYP4535′ and ‘Bussard’ were identified as the most resistant (4.5%) and most susceptible (22.9%) parental line to leaf rust, respectively. The analysis of variance showed significant differences concerning genotype, environment, and the interaction between genotype and environment (Table 2).

**Table 1:** Descriptive statistics and heritability / repeatability for field trials (AO) and seedling test (IT and Pi)

Trait^a^	Mean founders	Mean population	Min^b^	Max^c^	SE_±_^d^	CV^e^	*h*^2^/*rep*
AO [%]	13.70	13.50	0	63.75	0.17	0.83	0.83^f^
IT [1–10]	3.32	3.84	0	10.00	0.06	0.96	0.93^g^
Pi [%]	8.06	8.47	0	57.73	0.18	0.72	0.91^g^

^a^ Average ordinate (AO), infection type (IT), infected leaf area (Pi)

^b^ Minimum

^c^ Maximum

^d^ Standard error

^e^ Coefficient of variance

^f^ Broad-sense heritability (*h*^2^)

^g^ Repeatability (*rep*)

**Table 2:** Analysis of variance of log_10_-transformed data for leaf rust severity evaluated in field trials (AO) and seedling test (IT and Pi)

Trait^a^/factor	DF^b^	*F* value	*P* value
**AO**			
Genotype	402	18.98	< 0.0001
Environment	4	16.05	0.0049
Genotype × environment	1605	2.39	< 0.0001
**IT**			
Genotype	402	17.69	< 0.0001
Replication	4	0.94	0.5196
**Pi**			
Genotype	402	16.63	< 0.0001
Replication	4	6.66	0.0426

Significance level at *P* < 0.05

^a^ Average ordinate (AO), infection type (IT), infected leaf area (Pi)

^b^ Degrees of freedom

Scoring qualitative resistance in seedling test was performed twice—using an image analysis software to obtain the Pi and visually by assessing the IT (1–10). For both traits, phenotypic data revealed a large variability (Fig. 1b and c). The average IT ranged from 0.1 to 9.2 (mean 3.8). For Pi, the disease severity was on average between 0 and 28.3% (mean 8.5%). Phenotypic distributions of IT and Pi were slightly bimodal, with 131 and 185 RILs showing IT values smaller 2 (few areas with restricted sporulation) and Pi values below 5%, respectively. Maximal scores of 10 (IT) and 57.7% (Pi) were observed (Table 1). The population means of IT and Pi were not significantly different from the means of parental lines. According to the results of field trials, ‘BAYP4535’ and ‘Ambition’ were the most resistant and susceptible founders, respectively, in the seedling inoculation test. Pearson correlation displayed a high correlation coefficient between both traits (*r* = 0.91; Fig S2 C). The qualitative traits IT and Pi and the quantitative scoring of AO showed weak correlations of *r* = 0.27 and *r* = 0.24 (Fig S2 A and B). For both traits, a significant genotype effect was observed, while for Pi also a significance of replication was found. Repeatability of both traits was high with *rep*(IT) = 0.93 and *rep*(Pi) = 0.91 (Table 1). From the parental lines, only ‘BAYB4535 showed all stage resistance, whereas cv. ‘Event’, Format’, ‘Julius’, ‘Potenzial’ only showed resistance at seedling and ‘Firl3565’ at adult plant stage, respectively. In total, 68 genotypes in the population expressed all stage resistance, 92 genotypes showed resistance only at seedling stage and 44 genotypes were observed showing APR.

### QTL mapping

Overall, SIM revealed 19 QTL located on chromosomes 1A, 4A, 4D, 5A, 6B, 7A, and 7D. Hence, five QTL were detected based on field data and seven QTL for seedling resistance, each for IT and Pi values (Table 3, Tab. S2).

**Table 3:** QTL for resistance to *Puccinia triticina* in the BMW population detected in field trials and seedling tests

Trait	Chr.^a^	Pos.[cM]^b^	SI [cM]^c^	*P* value	*R*^2d^	No. Env.^f^	Eff (A)^g^	Eff (B)^g^	Eff (C)^g^	Eff (D)^g^	Eff (E)^g^	Eff (F)^g^	Eff G)^g^	Eff (H)^g^
**AO**	4A	133	125–151	2.00E-22	0.31	4	− 0.17	− 3.12	+ 0.93	− 1.21	+ 0.90	+ 0.88	+ 0.86	+ 0,83
	4A	172	170–174	2.52E-58	0.50	4	+ 0.94	− 3.96	− 0.13	− 0.44	+ 2.04	− 0.18	+ 1.94	− 0,23
	6B	22	10–30	1.49E-05	0.08	1	+ 0.16	− 1.23	+ 1.70	+ 1.25	− 1.32	− 0.89	− 1.16	+ 1.47
	7A	368	346–379	1.52E-05	0.07	1	− 0.16	− 1.26	+ 1.10	− 1.39	− 1.42	+ 1.22	+ 0.94	+ 0.89
	7D	18	15–19	3.68E-32	0.28	4	na	− 3.16	na	na	+ 1.44	+ 0.94	+ 0.18	+ 0.58
**IT**	1A	28	0–34	1.55E-06	0.11		na	+ 0.76	− 0.27	na	− 0.75	− 0.98	− 0.67	+ 1.88
	4A	170	168–174	8.79E-23	0.28		0.00	− 2.57	− 1.32	+ 1.59	+ 1.15	− 1.10	+ 1.58	+ 1.12
	4D	69	59–86	2.57E-05	0.01		na	+ 0.01	na	na	+ 1.16	na	na	-1.98
	5A	112	102–152	1.56E-05	0.08		− 0.99	+ 0.21	+ 0.63	+ 0.06	− 2.06	+ 0.67	+ 0.87	+ 0.60
	5A	139	99–152	3.31E-05	0.05		− 0.26	+ 0.78	+ 1.29	− 1.29	− 1.44	+ 1.12	− 1.29	+ 1.07
	6B	249	248–250	2.18E-55	0.01		− 0.5	na	na	na	na	na	na	+ 0.5
	7D	22	15–30	6.14E-12	0.17		na	− 1.84	na	na	+ 0.61	+ 0.61	+ 0.02	+ 0.61
**Pi**	1A	26	0–34	8.11E-06	0.12		na	+ 0.60	+ 1.64	na	− 1.35	− 1.59	− 1.27	+ 1.98
	4A	171	168–174	1.11E-16	0.21		+ 0.33	− 4.14	− 1.33	+ 1.67	+ 1.47	− 0.88	+ 1.47	+ 1.42
	4D	72	59–86	4.27E-06	0.09		na	− 0.06	na	na	+ 1.6	na	na	− 1.54
	6B	249	247–250	1.76E-91	< .01		− 0.52	na	na	na	na	na	na	+ 0.53
	7A	65	54–87	7.02E-06	0.05		na	+ 1.05	+ 2.15	− 0.95	− 0.13	− 0.73	− 1.41	+ 0.03
	7A	99	94–111	6.12E-06	0.08		− 0.97	+ 1.38	+ 0.81	+ 0.08	+ 0.21	+ 0.23	− 2.38	+ 0.65
	7D	22	15–30	5.64E-09	0.14		na	− 2.78	na	na	+ 1.11	+ 1.11	− 0.27	+ 0.85

^a^ Chromosomal position of QTL

^b^ Position of peak marker based on Stadlmeier et al. (2018)

^c^ Support interval

^d^ Proportion of phenotypic variance explained by a single QTL

^f^ Number of single environments in which QTL was detected

^g^ Additive effects ( ±) of the founders Event (**A**), BAYP4535 (**B**), Ambition (**C**), Firl3565 (**D**), Format (**E**), Potenzial (**F**), Bussard (**G**) and Julius (**H**) relative to the population mean. Shown values are back-transformed to the original trait scale

Founder effects were reported as not available (na) if none of the RILs reached the probability threshold of 0.7

The phenotypic variance (*R*^2^) explained by the individual QTL detected in field trials ranged between 8 and 50%, with support intervals (SI) from 4 to 33 cM. The two strongest QTL, explaining 31% and 50% of *R*^2^, were located on chromosome 4A with peak markers at 133 cM and 172 cM. The largest allelic effects of these QTL were contributed by ‘BAYP4535’, reducing disease severity by 3.1% and 4.0%, respectively. Another QTL detected on chromosome 7D (at 18 cM) explained 28% of the phenotypic variance with ‘BAYP4535′ as the most resistant founder, reducing infected leaf area by 3.2%. Remaining QTL on chromosomes 6B (at 22 cM) and 7A (at 368 cM) accounted for 8% and 7% of leaf rust variation. For these QTL, cv. ‘Format’ contributed the largest allelic effect reducing infected leaf area by 1.3% and 1.4%, respectively.

For IT, phenotypic variance explained by the seven QTL ranged from 1 to 28% with SIs ranging between 2 and 53 cM (Table 3). QTL on chromosomes 4A and 7D accounted for the highest *R*^2^ i.e. 28% and 17% with peak markers at 170 cM and 22 cM. The largest allelic effect of both QTL was contributed by ‘BAYP4535′, reducing disease severity by 2.6 and 1.8 scores, respectively. On chromosome 1A, one QTL was detected at 28 cM, explaining 11% of the phenotypic variance. A maximum effect of -1.0 score was detected for cv. ‘Potenzial’. Furthermore, two QTL were detected on chromosome 5A with 8% (at 112 cM) and 7% (at 139 cM) of the explained variance. SIs of these QTL ranged from 102 to 152 cM and from 99 to 152 cM, respectively. For both, ‘Format’ contributed the highest allelic effect (− 2.1 and − 1.4 scores). QTL located on chromosomes 4D (69 cM) and 6B (249 cM) explained only 1% of the phenotypic variance, each. By analyzing each environment separately, the two QTL on chromosomes 4A were also detected in LEN18, QLB17, QLB18 and SOE18, as well as LEN18, QLB18, SOE17 and SOE18, respectively. The QTL on chromosome 6B and 7D was detected in one (SOE18) and four (LEN18, QLB17, QLB18, SOE18) environments.

SIM of Pi values also revealed seven individual QTL with *R*^2^ ranging from less than 1% to 21%. The support intervals varied between 2 and 34 cM. QTL regions on chromosomes 1A, 4A, 4D, 6B and 7D overlapped with QTL regions detected for IT (Table 3). Nevertheless, smaller *R*^2^ of 21% (4A), 14% (7D) and < 1% (6B), as well as larger *R*^2^ of 12% (1A) and 9% (4D) were calculated for individual QTL. The maximal reducing effect of the QTL on chromosomes 1A, 4A, 4D, and 6B ranged between 0.5% and 4.1%, while for 7D, only the founder ‘BAYP4535′ showed a reducing allelic effect of 2.8%. Additionally, two QTL were detected on chromosome 7A at 65 cM and 99 cM, accounting for 5% and 8% of the phenotypic variance. SI ranged from 54 to 87 cM and from 94 to 111 cM, respectively. Founders ‘Firl3565′ and ‘Bussard’ contributed the largest allelic effect, reducing the disease severity by 0.9% and 2.4%.

Based on support intervals of 19 QTL, detected in total for the different traits, 11 main QTL were identified (Fig. S3, Table 4). In silico annotations of peak markers revealed seven genes with known functions partly involved in resistance. Hence, marker *CAP8_c2448_355* on chromosome 1A referred to a DnaJ domain. A Protein kinase domain and a NB-ARC domain were identified for peak markers of *QLr.jki-4A.1* and *QLr.jki-4A.2* on chromosome 4A. Marker *AX-*95126745 on chromosome 4D and *RAC875_c31670_*389 on chromosome 5A referred to a cation/calcium exchanger 4 and ankyrin repeats, respectively. For peak markers of *QLr.jki-7A.1* and *QLr.jki-7A.1* on chromosome 7A, a pyridoxal-phosphate dependent enzyme and a sugar efflux transporter were annotated, respectively.

**Table 4:** Main QTL for resistance to *Puccinia triticina* merged over all evaluated traits

QTL	Chr.^a^	Peak marker	Pos.[cM]^b^	SI [cM]^c^	Pos.RefSeq [bp]^d^		Potential origin	Adjacent *T.aestivum* gene	Orthologous gene	Identity	Functional annotation
					Start	End					
*QLr.jki-1A.1*	1A	CAP8_c2448_355	27.63	0–34	10069841	10069932	Potenzial	TraesCS1A01G020600	TRIUR3 04361^e^	93.63	DnaJ domain
		RAC875_c57939_78	26.12		11571831	11571931		TraesCS1A01G023400	F775 01617^f^	94.93	
*QLr.jki-4A.1*	4A	Kukri_rep_c109167_89	133.99	125–151	634737614	634737686	BAYP4535	TraesCS4A01G361100	TRIUR3 34719^e^	99.78	Protein kinase domain
									F775 31833^f^	99.28	
*QLr.jki-4A.2*	4A	BobWhite_c47168_598	171.04	168–174	726214891	726214991	BAYP4535	TraesCS4A01G461700			NB-ARC domain
		Excalibur_c46904_84	169.52		737340474	737340573		TraesCS4A01G481400	TRIUR3 03302^e^	96.59	
									F775 10262^f^	96.25	
*QLr.jki-4D.1*	4D	BS00023112_51	69.43	58–86	455763978	455764078	Julius	TraesCS4D01G285000	F775 08229^f^	100.00	
		AX-95126745	71.96		464988433	464988533		TraesCS4D01G294600	F775 05351^f^	99.69	Cation/calcium exchanger 4
*QLr.jki-5A.1*	5A	IAAV2363	111.77	98–152	481901324	481901524	Format	TraesCS5A01G271500	F775 15669^f^	96.01	
		RAC875_c31670_389	138.69		514094550	514094650		TraesCS5A01G305200	F775 21555^f^	98.40	Ankyrin repeats
*QLr.jki-6B.1*	6B	AX-94557244	21.83	10–30	25914587	25914687	Format	TraesCS6B01G041900			
*QLr.jki-6B.2*	6B	RAC875_c57692_88	249.34	247–250	712673112	712673182	Event	TraesCS6B01G456500			
*QLr.jki-7A.1*	7A	BS00011330_51	64.66	54–87	63112744	63112844	Firl3565	TraesCS7A01G102800	TRIUR3 02989^e^	99.72	Pyridoxal-phosphate dependent enzyme
									F775 27910^f^	95.24	
*QLr.jki-7A.2*	7A	wsnp_Ku_c26530_36497050	98.82	93–111	84772316	84772460	Bussard		TRIUR3 06012^e^	94.36	
*QLr.jki-7A.3*	7A	BS00011622_51	368.31	346–379	712309001	712309084	Format	TraesCS7A01G533900	TRIUR3 33918^e^	88.99	Sugar efflux transporter for intercellular exchange
									F775 06947^f^	87.19	
*QLr.jki-7D.1*	7D	AX-94930280	18.13	15–30	16119641	16119741	BAYP4535	TraesCS7D01G030600			
		IACX11794	21.65		12470235	12470390		TraesCS7D01G026100	F775 15174^f^	97.59	

^a^ Chromosomal position of QTL

^b^ Position of peak marker based onStadlmeier et al. (2018)

^c^ Support interval

^d^ Position of peak marker at the reference genome RefSeq v1.0

^e^ *Triticum urartu*

^f^ *Aegilops tauschii*

## Discussion

Continuous evolution of leaf rust results in the emergence of new pathotypes virulent against single major resistance genes commonly present in cultivars. Many of these race specific *Lr* genes have been broken down in the past (Kolmer 2005; Serfling et al. 2013). Detection of effective leaf rust resistances is of essential importance to avoid rust epidemics. Therefore, experimental populations such as MAGIC populations provide powerful tools to discover, characterize, and deploy QTL for complex traits including resistances (Cavanagh et al. 2008). Out of 80 designated *Lr* genes, it was reported, that only *Lr1*, *Lr3*, *Lr10*, *Lr13*, *Lr14a*, *Lr17b*, *Lr20*, *Lr24*, *Lr26*, *Lr34*, and *Lr37* were used individually or in combination in European varieties (Goyeau et al. 2006; Goyeau and Lannou 2011; Serfling et al. 2013). The BMW population emerged from crosses of eight elite parental lines originating from Germany and Denmark. Nevertheless, Stadlmeier et al. (2018) were able to show the potential of the BMW population to detect new QTL for resistance to powdery mildew, septoria tritici blotch, as well as tan spot, and in general the usefulness for further gene mapping studies (Stadlmeier et al. 2018, 2019).

In this study, phenotyping of 394 RILs from the BMW population resulted in a broad variability of resistance to *Puccinia triticina*. Despite an average correlation coefficient of 0.54 between the disease severities in five environments, a broad sense heritability of 0.83 was calculated which is in the range of previously published studies (Bemister et al. 2019; Gao et al. 2016; Zhang et al. 2017, 2019). This may hint to a quantitative inheritance due to QTL involved in slow rusting loci, which are characterized by relatively high heritabilities (Kolmer 1996). Phenotypic distribution for field trials was slightly right-skewed, while almost a bi-modal distribution was observed for both IT and Pi values in seedling test. This may give hint that mostly horizontal (quantitative) or vertical (qualitative) resistances were scored, respectively. Calculation of correlation between field data and seedling test results showed *r* values of 0.27 (IT) and 0.24 (Pi), which are in accordance with correlations reported by Gao et al. (2016). Different virulence/avirulence patterns of leaf rust races may be an explanation for these low correlations (Gao et al. 2016). While a single highly aggressive race, with many virulence genes was used for artificial inoculation for seedling tests and field trials in QLB, field trials in SOE and LEN were conducted under natural infection pressure.

Overall, simple interval mapping detected 19 QTL, which corresponded to 11 distinct chromosomal regions (Table 4, Fig. S3). QTLs identified using the LSmeans over all environments were also identified by analyzing the single environments separately. Out of the 11 distinct chromosomal regions three QTL were detected at the adult plant stage. Six QTL conferred seedling resistance and two were active in both growth stages, indicating the presence of effective all-stage leaf rust resistance genes. In total, the regions were located on wheat chromosomes 1A, 4A, 4D, 5A, 6B, 7A and 7D. Peak markers of QTL could be partially annotated to genes, known to be involved in quantitative resistances to leaf rust, e.g. sugar efflux transporters, DnaJ domain belonging to heat shock protein family (Bekh-Ochir et al. 2013), a protein kinase domain, a NB-ARC domain and a cation/calcium exchanger. Such genes show an increased expression during defense reactions in wheat-leaf rust (Sharma et al. 2018) and wheat-stripe rust interactions (Wang et al. 2020) and as response to environmental stresses.

In this study, *QLr.jki-1A.1* on chromosome 1A is based on the evaluation of IT and Pi in seedling tests and is physically located in a region between 1.3 Mbp and 12.5 Mbp (Table 5). Pinto da Silva et al. (2018) reviewed 11 QTL described in hexaploid wheat located on chromosome 1A. Based on available physical marker positions*, QLr.ccsu-1A.1* and *QLr.cau-1AS* identified in two different studies, were found to correspond to the region of *QLr.jki-1A.1* (Du et al. 2015; Kumar et al. 2013). While *QLr.ccsu-1A.1* is only 1.7 Mbp and 0.2 Mbp apart from our peak markers, the distance of the linked marker to *QLr.cau-1AS* is 2.4 Mbp and 3.9 Mbp, respectively (Tables 4, 5). Additionally, Elbasyoni et al. (2017) detected several marker-trait associations (MTAs) covering a region from 7.2 Mbp to 13.7 Mbp, which includes the region of *QLr.jki-1A.1*. Furthermore, the resistance gene *Lr10*, which is completely sequenced, is mapped at 12.6 Mbp, i.e. 2.5 Mbp and 1 Mbp apart from our peak marker (Table 4; Feuillet et al. 1997, 2003). Thus, and due to the fact that *Lr10*, *Lr1*, *Lr3a* and *Lr20* are the most prevalent genes used worldwide, *Lr10* is a promising candidate for the QTL aforementioned (Dakouri et al. 2013).

**Table 5:** Comparison of physical positions of the QTL identified in the present study (bold) with those reported previously. Physical positions based on comparison of marker sequence data to the wheat reference genome (RefSeq1.0)

QTL	Marker interval	Physical position [Mbp]	Genetic material	References
***QLr.jki-1A.1***	IAAV3919–Tdurum_contig42479_3800	1.3–12.5	BMW population (RIL^a^)	*Lr10*?
*QLr.ccsu-1A.1*	Xbarc263–Xcdo426	11.8–na^b^	Opata85 × W-7984 (RIL)	Kumar et al. (2013)
*QLr.cau-1AS*	gpw2246	7.7	Luke × AQ24788-83 (RIL)	Du et al. (2015)
*MTA*	IWA3182–IWA7191	7.1–13.7	Spring wheat collection	Elbasyoni et al. (2017)
*Lr10*		12.6		Feuillet et al. (2003)
***QLr.jki-4A.1***	AX-95253498–TA006348.0950	618.6–649.9	BMW population (RIL)	
*MTA*	IWA2816	641.5	Hexaploid Wheat Landraces	Kertho et al. (2015)
***QLr.jki-4A.2***	Tdurum_contig75819_1220–Excalibur_c33542_113	712.9–na	BMW Population (RIL)	
*4A_t2*	BobWhite_c47168_289	726.2	Elite spring wheat lines	Gao et al. (2016)
*QLr.hebau-4AL*	BobWhite_c15697_675–Excalibur_c2827_580	598.7–726.4	Zhou8425B × Chinese Spring (RIL)	Zhang et al. (2017)
***QLr.jki-4D.1***	AX-94793903–AX-94838884	130.9–479.7	BMW population (RIL)	Novel?
*QLr.fcu-4DL*	Xgdm61–Xcfa2173	na	TA4152-60 × ND495 (DH^c^)	Chu et al. (2009)
*QLr.hebau-4DL*	AX-110476142–AX-111092299	381.2–428.6	Pingyuan50 × Mingxian169	Zhang et al. (2019)
*QLr.sfrs-4DL*	Xglk302b–Xpsr1101a	na	Forno × Oberkulmer (RIL)	Messmer et al. (2000)
*Lr67*	Xgwm165–Xgwm192	412.7	RL6077 × Avocet (RIL)	Herrera-Foessel et al. (2011)
***QLr.jki-5A.1***	AX-94732470–wsnp_Ex_c49211_53875600	444.6–na	BMW population	Novel?
*QLr.cim-5AC*	wPt-3187–wPt-7769	Na–464.7	Avocet-YrA × Kenya Kongoni (RIL)	Calvo-Salazar et al. (2015)
***QLr.jki-6B.1***	AX-94739546–TA003005.0339	19.3–34.3	BMW population	Novel?
*QLr.caas-6BS.1*	Xcfd13–Xwmc487	34.2–36.5	Bainong64 × Jingshuang16 (DH)	Ren et al. (2012)
*QLr.wpt-6BS.2*	wPt2175	na^b^	Winter wheat accessions	Gerard et al. (2018)
***QLr.jki-6B.2***	wsnp_Ex_c54772_57528275–Excalibur_c29748_954	710.1–719.7	BMW population	*Lr3*?
*QLr.cim-6BL*	277,143–1,234,305	714.3–na	Bairds × Atred#1 (RIL)	Lan et al. (2017)
*6B_4*	BobWhite_c43263_180–BS00011795_51	718.9–720.6	Elite spring wheat lines	Gao et al. (2016)
***QLr.jki-7A.1***	BobWhite_rep_c58252_112–wsnp_BF473884A_Ta_1_3	54.9–71.1	BMW population	Novel
***QLr.jki-7A.2***	RAC875_c75528_355–BS00024786_51	79.6–na	BMW population	Novel?
*QLr.stars-7AS1*	wsnp_Ex_c41150_48040078	78.4	Winter wheat accessions	Li et al. (2016)
*MTA*	IWA7192	81.1	Spring wheat collection	Elbasyoni et al. (2017)
*Lr47*		115		Helguera et al. (2000)
***QLr.jki-7A.3***	Tdurum_contig29240_206–wsnp_CAP11_c298_250917	702.4–724.1	BMW Population	*Lr20*?
*MTA*	IWA4175	717.1	Spring wheat accessions	Turner et al. 2017
***QLr.jki-7D.1***	TA016282.1180–AX-94883448	na–29.4	BMW Population	Novel
*Lr34*		47.4–51		Krattinger et al. (2009)

^a^ Recombinant inbred line population

^b^ marker information was not available or position could not be identified in the RefSeq v1.0

^c^ Doubled haploid population

On chromosome 4A, two regions harboring leaf rust resistance were identified in this study (*QLr.jki-4A.1*, *QLr.jki-4A.2*, Table 4). To date, there are two *Lr* genes, *Lr28* originating from *Ae. speltoides* and *Lr30* from *T. aestivum*, and two QTL reported on chromosome 4A (Dyck and Kerber 1971; McIntosh et al. 2013; Pinto da Silva et al. 2018). Kertho et al. (2015) found one MTA at 641.5 Mbp, using the leaf rust race MCDL. Therefore, the marker is physically located within the region of *QLr.jki-4A.1*, but 6.8 Mbp apart from our peak marker. Due to the specific virulence pattern of the MCDL race, which is avirulent to *Lr30*, the MCDL-MTA might identify this *Lr* gene. However, to our knowledge, no mapping information for *Lr30* is available to allow a more precise comparison between *Lr30*, the MCDL-MTA and *QLr.jki-4A.1* detected in this study. Another significant MTA (*4A_t2*, Gao et al. 2016) was detected in the region of *QLr.jki-4A.2,* only 309 bp apart from the peak marker for this QTL (Table 4). *4A_t2* was mapped approximately at the position of the marker linked to *Lr28* (Bipinraj et al. 2011). This may be a hint that *QLr.jki-4A.2* also corresponds to *Lr28*, but further analyses have to be conducted. Furthermore, Zhang et al. (2017) reported a minor QTL for APR in Chinese Spring (*QLr.hebau-4AL*), which is physically located between 598.7 Mbp and 726.4 Mbp. This region includes both QTL on chromosome 4A detected in this study (Table 5).

In total, nine QTLs were detected on chromosome 4D so far, including the resistance gene *Lr67/Yr46/Sr55* (Herrera-Foessel et al. 2011; McIntosh et al. 2013; Pinto da Silva et al. 2018). In this study, *QLr.jki-4D.1* was detected for both IT and Pi in the seedling tests and mapped at the distal end of chromosome 4DL. Physically, it is located in a large interval from 130.9 Mbp to 479.7 Mbp (Table 5) with peak markers at 455.8 Mbp and 465 Mbp, respectively (Table 4). Chu et al. (2009) located a QTL (*QLr.fcu-4DL*) in douple-haploid population ‘TA4152-60 × ND495′, mapped at a similar position as *Lr67*, around 412.7 Mbp (Herrera-Foessel et al. 2011; Zhang et al. 2019). Another QTL on chromosome 4DL (*QLr.hebau-4D*) was located between 381.2 Mbp and 428.6 Mbp (Zhang et al. 2019). Considering the physical distances to our peak marker, it appears that *QLr.jki-4D.1* is independent from *QLr.fcu-4DL*, *QLr.hebau-4D,* and *Lr67* (Table 5). A higher similarity may exist with another QTL (*QLr.sfrs-4DL*) detected by Messmer et al. (2000). This QTL resulted in an APR and was mapped in the Swiss RIL population ‘Forno × Oberkulmer’ also at the distal end of chromosome 4DL. Since *QLr.jki-4D.1* has only been detected at the seedling stage, *QLr.sfrs-4DL* also seems to be located in a different region and with the available data, it is not possible to further determine whether it corresponds to our regions.

On chromosome 5A one QTL (*QLr.jki-5A.1*) was detected in seedling tests for IT (Table 4). To our knowledge, on chromosome 5A there is no designated *Lr* gene and only two QTL (*QLr.cim-5AC*, *QLr.cimmyt-5A*) are known (Calvo-Salazar et al. 2015; Rosewarne et al. 2012). *QLr.cimmyt-5A* was mapped on the long arm of chromosome 5A, closely linked to *Vrn-A1* at 587.0 Mbp (Rosewarne et al. 2012). *QLr.cim-5AC* was located in the centromeric region of chromosome 5A and flanked by markers *wPt-7769* and *wPt-3187*, of which the latter is located at 464.7 Mbp (Table 5). When comparing the physical positions of these three QTL, it is more likely that *QLr.jki-5A.1* corresponds to *QLr.cim-5AC* or is a novel QTL.

On chromosome 6B, two QTL were identified (*QLr.jki-6B.1* and *QLr.jki-6B.2*) in the present study (Table 4). *QLr.jki-6B.1* was mapped on the short arm of chromosome 6B, at 19.3—34.3 Mbp (Table 5). Up to now, 5 QTL have been described on chromosome 6BS, but only *QLr.caas-6BS.1,* derived from the wheat cultivar Bainong 64, was physically localized in the region between 32 and 34 Mbp (Gerard et al. 2018; Kankwatsa et al. 2017; Ren et al. 2012). Gerard et al. (2018) stated that another QTL (*QLr.wpt-6BS.2*) is genetically located in the same region as *QLr.caas 6BS.1*, whereas *QLr.wpt-6BS.2* was mapped close to the centromere, a region clearly distinct from *QLr.jki-6B.1* (Table 5). Therefore, further studies are required to confirm whether our QTL is located closely to these known QTL. The second QTL *QLr.jki-6B.2* was mapped at the distal end of chromosome 6BL, within a small interval encompassing 247 cM to 250 cM (710 – 720 Mbp). Out of six QTL already detected on chromosome 6BL, two QTL (*QLr.cim-6BL* and *6B_4*) were also located at the distal end of chromosome 6BL (Chu et al. 2009; Gao et al. 2016; Lan et al. 2017; Rosewarne et al. 2012; William et al. 2006). The DArTseq markers *1234305* and *2277143* flank *QLr.cim-6BL* detected by Lan et al. (2017). Marker *2277143* was converted into a diagnostic KASP marker, which is located at 714.3 Mbp, i.e. 1.6 Mbp distal from our peak marker of *QLr.jki-6B.2* (Tables 4 , 5 ). The results of Lan et al. (2017) indicated uniqueness of *QLr.cim-6BL*, showing no relationship to other QTL on chromosome 6BL, as well as to *Lr3a* co-segregating with *Xmwg798* (Sacco et al. 1998)*.* However, the second known QTL *6B_4* was physically mapped between 718.9 Mbp and 720.6 Mbp, and appeared to be in high linkage disequilibrium with *Lr3* (Gao et al. 2016). Regarding the similar physical regions, *QLr.jki-6B.2* may correspond to *QLr.cim-6BL* and *6B_4*, but further research is needed to come to a closer understanding of the relationship between these QTL and *Lr3.*

On chromosome 7A, the major resistance genes *Lr20,* forming a disease-resistance gene cluster with *Pm1,* and *Lr47*, which was transferred from chromosome 7S of *Ae. speltoides* have been reported (Dubcovsky et al. 1998; Neu et al. 2002). Additionally, three QTL on chromosome 7AL and several MTAs were detected (Pinto da Silva et al. 2018). In the present study, three QTL (*QLr.jki-7A.1* to *QLr.jki-7A.3*) were identified on chromosome 7A. The first two QTL were detected for Pi in the seedling test and their support intervals were separated from each other by a map distance of 7.1 cM on chromosome 7AS. *QLr.jki-7A.1* was physically mapped between 54.9 Mbp and 71.1 Mbp (Table 5). To our knowledge, no QTL have been reported in this region. Hence, *QLr.jki-7A.1* might be a novel QTL. The second QTL (*QLr.jki-7A.2*) on chromosome 7AS was located between 93 and 111 cM. The peak marker was mapped at 84.8 Mbp (Table 4). To date, there are two MTAs from different studies detected in similar regions as *QLr.jki-7A.2* (Elbasyoni et al. 2017; Li et al. 2016). The first MTA (*QLr.stars-7AS1*), associated with marker *IWA3760* was mapped at 78.4 Mbp, hence, it appears that *QLr.stars-7AS1* does not correspond to *QLr.jki-7A.2*. The second MTA (*IWA7192*) was detected by Elbasyoni et al. (2017) at 81.1 Mbp, and might be correspondent to the resistance gene *Lr47*. When comparing the physical position of a diagnostic marker for *Lr47* (around 115 Mbp), both *IWA7192*, and *QLr.jki-7A.2* seem to be different from this *Lr* gene (Helguera et al. 2000). Thus, *QLr.jki-7A.2* is likely a novel locus involved in resistance to *P. triticina*.

The third QTL (*QLr.jki-7A.3*) determined in field trials during this study was mapped between 346 and 379 cM on chromosome 7AL. This translates to a large physical distance between 702.4 Mbp and 724.1 Mbp, with the peak marker at 712.3 Mbp (Tables 4, 5). Out of five known regions on chromosome 7AL involved in leaf rust resistance (Kankwatsa et al. 2017; Li et al. 2016; Lu et al. 2017; Tsilo et al. 2014), only the MTA detected by Turner et al. (2017) may be localized within the region of *QLr.jki-7A.3*. The associated marker *IWA4175* was mapped at 717.1 Mbp, which is 4.8 Mbp apart from our peak marker*.* However, after Bonferroni correction, the marker was no longer significant (*P* < 0.1). The *Lr* gene *Lr20* is genetically located in the distal part of chromosome 7AL (Neu et al. 2002), which may correspond to *QLr.jki-7A.3*. Based on the available data, investigations with diagnostic markers need to be conducted to gain further insights.

Finally, one QTL was detected on chromosome 7DS, based on phenotypic data from field trials and seedling test (Table 4). To date, out of 21 QTL reported on chromosome 7D, 19 correspond to the resistance gene *Lr34*, which confers race non-specific, partial, and slow rusting resistance to leaf rust (Lagudah et al. 2009; Pinto da Silva et al. 2018). *Lr34* has been physically located at 47.4 Mbp (Krattinger et al. 2009). Thus, *QLr.jki-7D.1* identified in our study does not correspond to the resistance gene *Lr34* and the 19 QTL reported (Table 5). The remaining two QTL *QLr.cim-7DS* and *QLr.hebau-7DS* on chromosome 7DS, which were detected in the two RIL populations ‘Avocet-YrA × Francolin#1′ and ‘Shanghai3/Catbird × Naxos’, respectively, were located in different chromosome region (Lan et al. 2014; Zhou et al. 2014). Hence, *QLr.jki-7D.1* seems to be a novel locus.

The objective of this study was to identify QTL for resistance to leaf rust, using the Bavarian MAGIC Wheat population. We identified 19 leaf rust resistance QTL that were confined to 11 distinct chromosomal regions. To date, more than 249 leaf rust resistance QTL and 200 MTAs were reported covering all 21 chromosomes of hexaploid wheat (Pinto da Silva et al. 2018). These regions were identified in several mapping populations using different genotyping methods. Because of the absence of information on physical positions for many of these QTL, it is difficult to unequivocally determine the identity of newly described QTL. In the present study, six putatively new QTL were identified on chromosomes 4D, 5A, 6B, 7A and 7D. SNP markers linked to these regions may be converted into KASP markers suitable for MAS in wheat breeding programs (Neelam et al. 2013; Rasheed et al. 2016). This will enable stacking of the detected resistance loci to breed new varieties with an improved resistance to leaf rust.

## Electronic supplementary material

Below is the link to the electronic supplementary material.

**Fig. S1** Pearson correlation of leaf rust severity between different field trials. Diagonals are histograms of each environment (Lengern LEN 2018, Quedlinburg QLB 2017 2018, Söllingen SOE 2017 2018). *** denotes significance at α = 0.001. Lowess curves were adjusted to the data points with a smoothing range of 0.75, based on the ‘lowess’ function implemented in the R-based ‘stats’ package (JPEG 133 kb)

**Fig. S2** Pearson correlation (r) between averaged infection type (IT), infected leaf area (Pi) of seedling test and average ordinate (AO) of field trials (A, B), as well as correlation between IT and Pi (C). *** denotes significance at α = 0.001 (JPEG 78 kb)

**Fig. S3** Simple interval mapping of resistance to *Puccinia triticina* in field trials (A) and seedling test (B, C). The x axis shows the 21 wheat chromosomes. Positions are based on the genetic map, and the log_10_(p) values of each Marker are displayed on the y axis (black line). The red horizontal line represents the significance thresholds. SI of the significant QTL detected in this study are coloured in blue (JPEG 188 kb)

**Tab. S1** List of virulences and avirulences of *Puccinia triticina* isolate 77WxR used in field trials and seedling test. Brackets indicate ambiguous results due to the differing symptom ratings between replications or moderate susceptibility (based on Zetzsche et al. 2019) (DOCX 13 kb)

**Tab. S2** Complete information of the 19 QTL for leaf rust resistance in BMW population, evaluated in field trials (AO) and seedling test (IT and Pi) (XLSX 16 kb)
