# Supplementary figures and images for: QTL mapping of adult plant and seedling resistance to leaf rust (Puccinia triticina Eriks.) in a multiparent advanced generation intercross (MAGIC) wheat population
Source: Theor Appl Genet. 2020 Aug 19;134(1):37–51. doi: 10.1007/s00122-020-03657-2 (PMC7813716; doi:10.1007/s00122-020-03657-2)

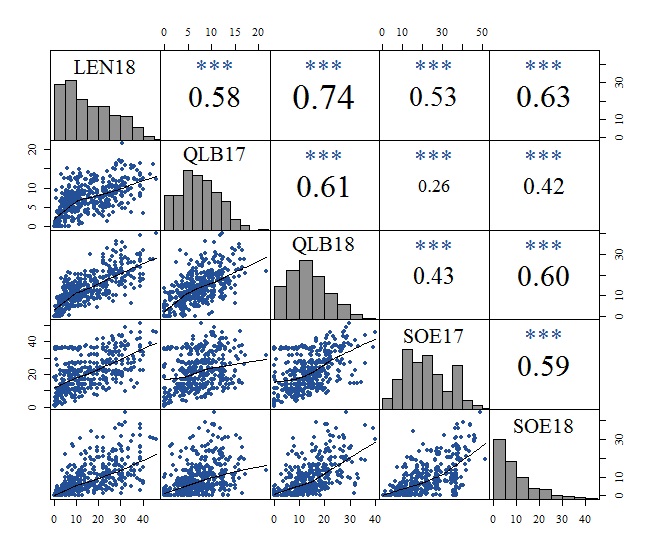

Supplement: Supplementary file 1 — Fig. S1 Pearson correlation of leaf rust severity between different field trials. Diagonals are histograms of each environment (Lengern LEN 2018, Quedlinburg QLB 2017 2018, Söllingen SOE 2017 2018). *** denotes significance at α = 0.001. Lowess curves were adjusted to the data points with a smoothing range of 0.75, based on the ‘lowess’ function implemented in the R-based ‘stats’ package (JPEG 133 kb) [file 122_2020_3657_MOESM1_ESM.jpg]

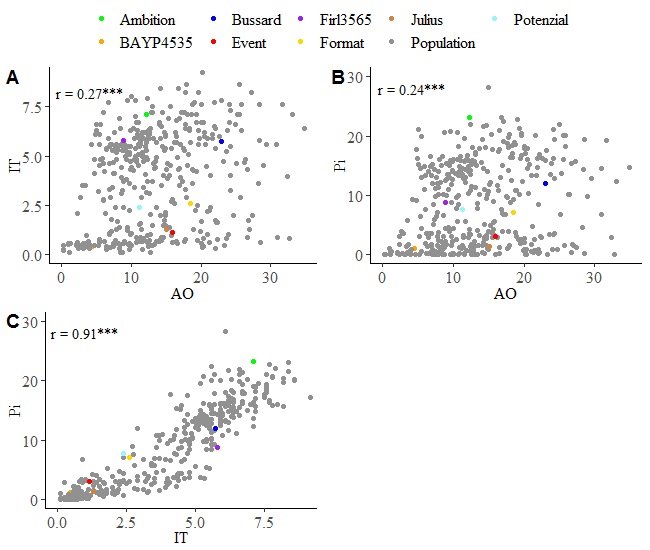

Supplement: Supplementary file 2 — Fig. S2 Pearson correlation (r) between averaged infection type (IT), infected leaf area (Pi) of seedling test and average ordinate (AO) of field trials (A, B), as well as correlation between IT and Pi (C). *** denotes significance at α = 0.001 (JPEG 78 kb) [file 122_2020_3657_MOESM2_ESM.jpg]

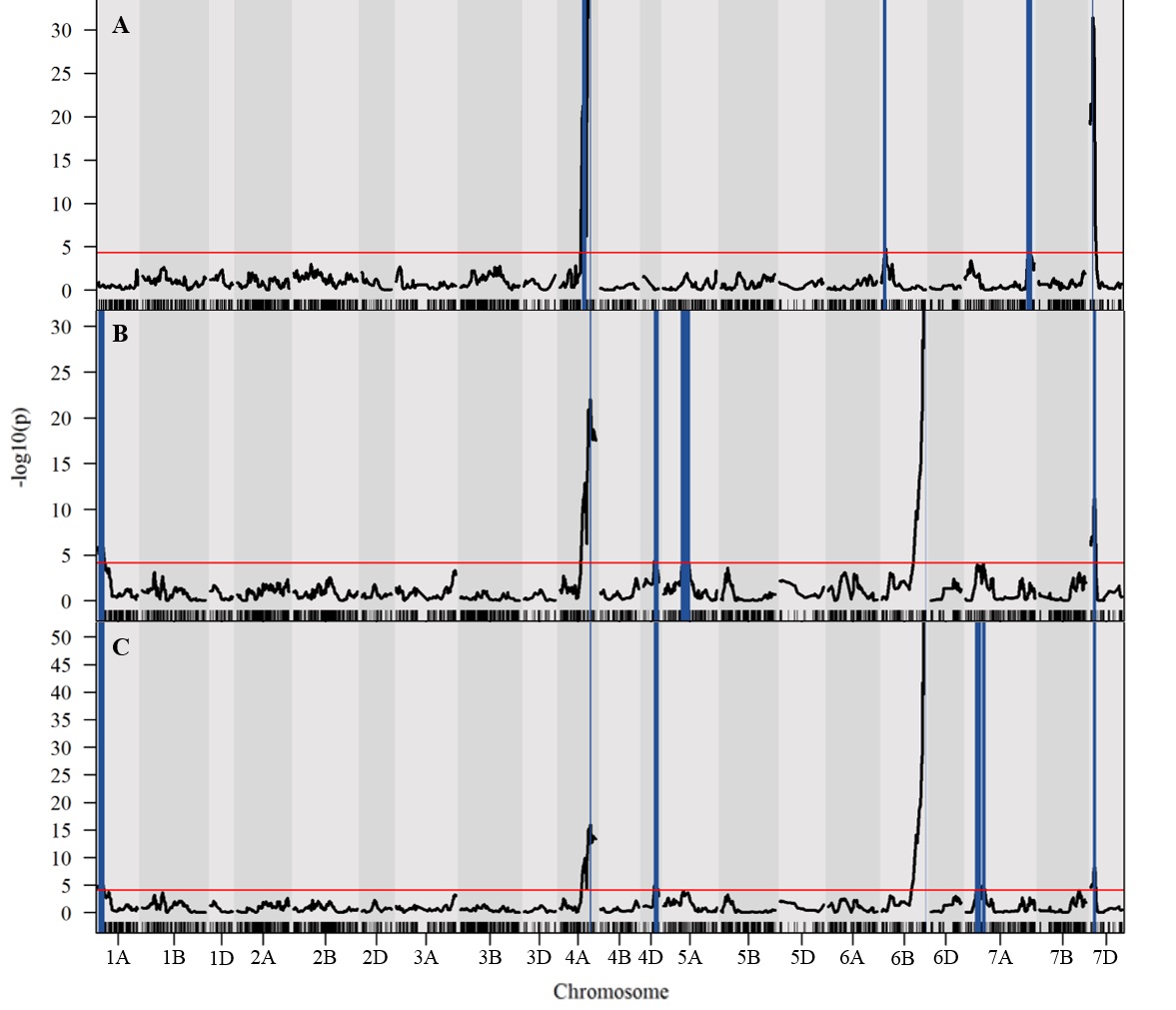

Supplement: Supplementary file 3 — Fig. S3 Simple interval mapping of resistance to Puccinia triticina in field trials (A) and seedling test (B, C). The x axis shows the 21 wheat chromosomes. Positions are based on the genetic map, and the log10(p) values of each Marker are displayed on the y axis (black line). The red horizontal line represents the significance thresholds. SI of the significant QTL detected in this study are coloured in blue (JPEG 188 kb) [file 122_2020_3657_MOESM3_ESM.jpg]
