# Supplementary material for: QTL mapping of adult plant and seedling resistance to leaf rust (Puccinia triticina Eriks.) in a multiparent advanced generation intercross (MAGIC) wheat population
Source: Theor Appl Genet. 2020 Aug 19;134(1):37–51. doi: 10.1007/s00122-020-03657-2 (PMC7813716; doi:10.1007/s00122-020-03657-2)
Supplement: Supplementary file 4 — Tab. S1 List of virulences and avirulences of Puccinia triticina isolate 77WxR used in field trials and seedling test. Brackets indicate ambiguous results due to the differing symptom ratings between replications or moderate susceptibility (based on Zetzsche et al. 2019) (DOCX 13 kb) [file 122_2020_3657_MOESM4_ESM.docx]

**Tab. S1** List of virulences and avirulences of Puccinia triticina isolate 77WxR used in field trials and seedling test. Brackets indicate ambiguous results due to the differing symptom ratings between replications or moderate susceptibility (based on Zetzsche et al. 2019)

| *P. triticina* | Virulence/avirulence | Virulences^b^ | Avirulences^b^ |
| --- | --- | --- | --- |
| 77WxR^a^ | 41/10 | *Lr1, Lr2a, Lr2b, Lr2c, Lr3a, Lr3bg, Lr3ka, Lr4, (Lr10), Lr11,* ***Lr12*****,* ***Lr13*****, Lr14a, Lr14b, Lr15, (Lr16), Lr17, Lr17b, Lr18, Lr20, (Lr21),* ***Lr22a*, Lr22b*****, Lr23, Lr26, (Lr28), (Lr30), (Lr32), Lr33,* ***Lr35*****, Lr36,* ***Lr37*****, Lr38, (Lr39), (Lr41), (Lr44), (****Lr46*****), (Lr48), Lr49, (Lr52), (LrB)* | *Lr9, Lr19, Lr24, Lr25, Lr27, Lr29,* ***Lr34*****, Lr45, Lr47, Lr53* |

^a^ isolated by Nover and Lehmann 1967, Collection Julius-Kühn-Feld Halle, Germany

^b^ tested for pathogenicity on differential Thatcher NILs carrying 51 different resistance genes *Lr1, Lr2a, Lr2b, Lr2c, Lr3a, Lr3bg, Lr3ka, Lr9, Lr10, Lr11, Lr12, Lr13, Lr14a, Lr14b, Lr15, Lr16, Lr17, Lr17b, Lr18, Lr19, Lr20, Lr21, Lr22a, Lr22b, Lr23, Lr24, Lr25, Lr26, Lr27 (=Lr31), Lr28, Lr29, Lr30, Lr32, Lr33, Lr35, Lr36, Lr37, Lr38 (Tc*6/TMR-514-12-24), Lr40, Lr41, Lr44, Lr45, Lr46, Lr47, Lr48, Lr49, Lr50, Lr51, Lr52, Lr53, LrB*

* Adult plant resistance (APR) *Lr* genes against leaf rust with known race specificity
